# Rapamycin regulates macrophage activation by inhibiting NLRP3 inflammasome-p38 MAPK-NFκB pathways in autophagy- and p62-dependent manners

**DOI:** 10.18632/oncotarget.17256

**Published:** 2017-04-19

**Authors:** Jung Hwa Ko, Sun-Ok Yoon, Hyun Ju Lee, Joo Youn Oh

**Affiliations:** ^1^ Department of Ophthalmology, Seoul National University Hospital, 03080, Seoul, Korea; ^2^ Laboratory of Ocular Regenerative Medicine and Immunology, Biomedical Research Institute, Seoul National University Hospital, 03080, Seoul, Korea; ^3^ R and D Laboratory, Eutilex Co., Ltd, 08594, Seoul, Korea

**Keywords:** autophagy, macrophage, NLRP3 inflammasome, p62/SQSTM1, rapamycin

## Abstract

Excessive and prolonged activation of macrophages underlies many inflammatory and autoimmune diseases. To regulate activation and maintain homeostasis, macrophages have multiple intrinsic mechanisms, one of which is modulation through autophagy. Here we demonstrate that autophagy induction by rapamycin suppressed the production of IL-1β and IL-18 in lipopolysaccharide- and adenosine triphosphate-activated macrophages at the post-transcriptional level by eliminating mitochondrial ROS (mtROS) and pro-IL1β in a p62/SQSTM1-dependent manner. In addition, rapamycin activated Nrf2 through up-regulation of p62/SQSTM1, which further contributed to the reduction of mtROS. Reduced IL-1β subsequently diminished the activation of p38 MAPK-NFκB pathways, leading to transcriptional down-regulation of IL-6, IL-8, MCP-1, and IκBα in rapamycin-treated macrophages. Therefore, our results suggest that rapamycin negatively regulates macrophage activation by restricting a feedback loop of NLRP3 inflammasome-p38 MAPK-NFκB pathways in autophagy- and p62/SQSTM1-dependent manners.

## INTRODUCTION

Macrophages are critical effectors of inflammation and the innate immune response. Upon tissue injury or infection, macrophages detect a wide range of endogenous and exogenous ‘danger’ signals and elicit the inflammatory process to protect the tissue [1, 2]. However, excessive or prolonged activation of macrophages leads to disease by causing collateral tissue damage and chronic para-inflammation [3]. Therefore, tight control of macrophage activation is pivotal to avoid tissue dysfunction and maintain homeostasis [4]. Indeed, there are multiple intrinsic mechanisms in macrophages to regulate activation, one of which is modulation by autophagy [5–7].

Autophagy is a ubiquitous eukaryotic process that enables cells to digest their cytoplasmic contents in lysosomes. Basal autophagy is necessary for cellular “housekeeping” to eliminate damaged organelles such as depolarized mitochondria through mitophagy [8–10]. Also, autophagy can be induced to preserve cellular homeostasis under diverse conditions of metabolic, physical, infectious, or immunologic stress [11, 12].

NLRP3 inflammasome is a multiprotein complex in myeloid cells including macrophages that mediates the cleavage of caspase-1, leading to the maturation and secretion of IL-1β and IL-18 [13]. Recently, studies have elucidated the role of autophagy in macrophage regulation through its effects on NLRP3 inflammasome activation. Blockade of autophagy enhanced IL-1β production in macrophages [14], whereas activation of autophagy inhibits IL-1β secretion by targeting ubiquitinated inflammasomes or pro-IL-1β for lysosomal degradation [14, 15]. Also, autophagy induction by inflammatory signals limits NLRP3 inflammasome activation by removing damaged mitochondria and preventing mitochondrial reactive oxygen species (mtROS) release [9, 10, 16, 17]. Furthermore, macrophages deficient in autophagic proteins ATG16L1, LC3B, or beclin 1 produce high levels of IL-1β and cleaved caspase 1 following stimulation by NLRP3 inflammasome activators [10, 18].

Rapamycin is the prototypical inhibitor of mechanistic target of rapamycin (mTOR) and inhibits mTOR complex 1 (mTORC1). The mTORC1 actively suppresses autophagy by phosphorylating ULK1 [6]. Therefore, rapamycin is a strong inducer of autophagy. In addition to autophagy modulation, mTORC1 signaling pathway regulates a variety of intracellular processes in innate immune cells through multiple mechanisms involving metabolism, protein translation, cytokine production, antigen presentation, macrophage polarization, or cell migration [6]. Given these complex functions of mTORC1, mTORC1 inhibition by rapamycin can be pro-inflammatory or anti-inflammatory depending on the cell types and environmental or cellular stress.

In this study, we investigated the effects of rapamycin on macrophages that were activated to trigger NLRP3 inflammasome. We demonstrate that autophagy induction by rapamycin inhibits IL-1β secretion in macrophages at the post-transcriptional level by reducing mtROS and pro-IL-1β. Moreover, rapamycin induces p62/SQSTM1 and Nrf2 (nuclear factor erythroid-derived-2-like 2) in an autophagy-independent manner, which helps further suppress mtROS and NLRP3 inflammasome. Diminished extracellular IL-1β subsequently reduces the transcriptional activity of IL1β-p38 MAP kinase (MAPK)-NFκB pathways. These results collectively suggest that rapamycin negatively regulates macrophage activation by augmenting autophagy and inhibiting a positive feedback loop of NLRP3 inflammasome-p38 MAPK-NFκB pathways.

## RESULTS

### Rapamycin inhibits NLRP3 inflammasome activation through autophagy induction

**Figure 1:**
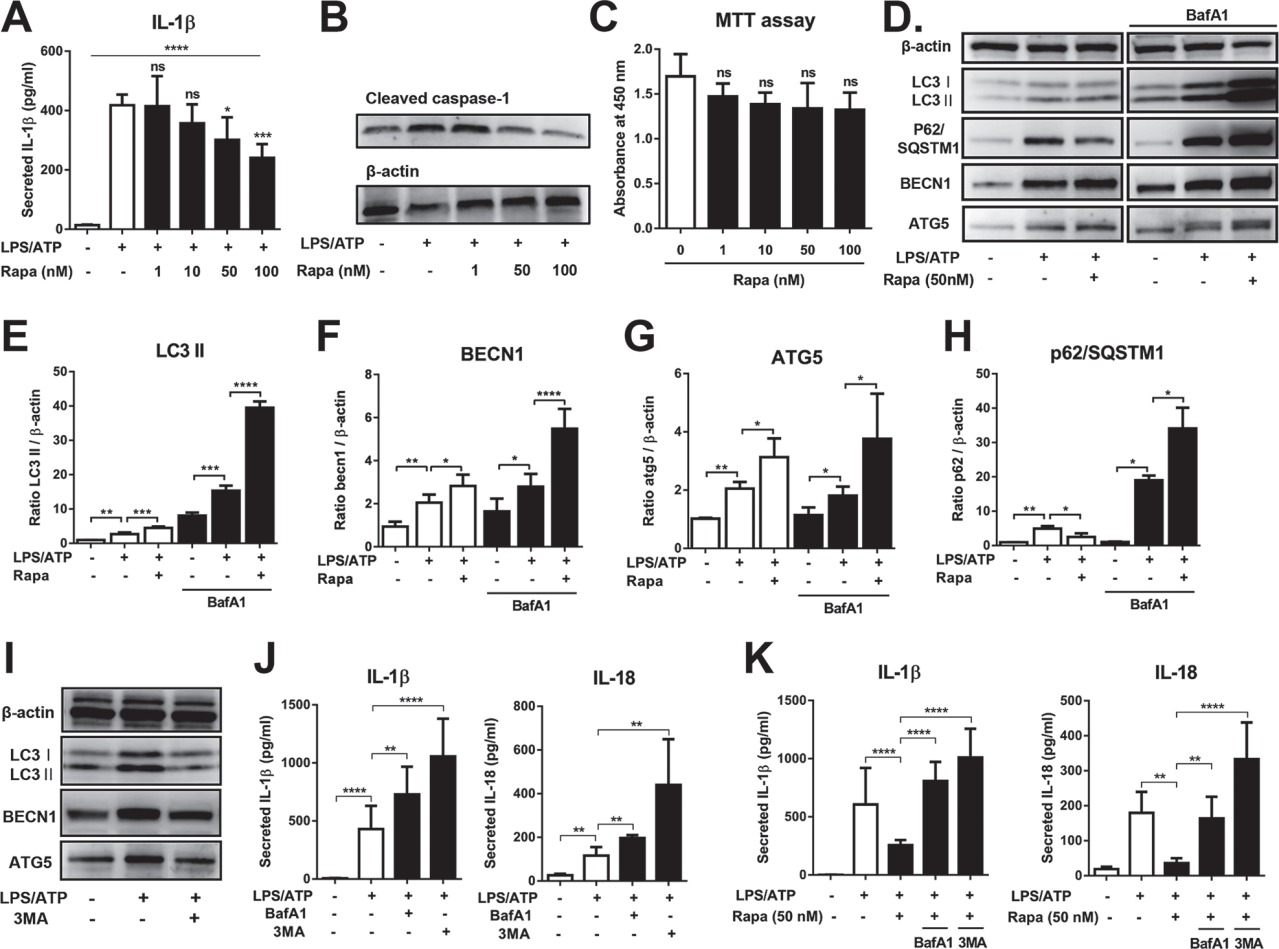
Rapamycin inhibits NLRP3 inflammasome activation through autophagy induction THP-1-differentiated macrophages were stimulated by LPS (2 μg/mL, 4 h), followed by ATP (5 mM, 45 min) in the presence of rapamycin (Rapa, 0 to 100 nM). After 18 h of culture, the cells and supernatants were analyzed. (**A**, **B**) The IL-1β secretion was quantified in supernatants by ELISA, and the level of cleaved caspase-1 measured in cell lysates by Western blot. (**C**) The cell viability was analyzed by MTT assay. (**D**) Representative images of Western blot analysis for LC3, p62/SQSTM1, beclin 1 (BECN1), and ATG5 in cell lysates from LPS/ATP-stimulated macrophages ± Rapa in the presence or absence of bafilomycin A1 (BafA1, 75 nM). (**E**–**H**) Densitometric analysis of the ratio of LC3 II, BECN1, ATG5, or p62/SQSTM1 relative to β-actin in (D). (**I**) Representative images of Western blot analysis for LC3, BECN1, and ATG5 in cell lysates from LPS/ATP-stimulated macrophages in the presence or absence of 3-methyl adenine (3MA, 2.5 mM). (**J**) ELISA analysis of IL-1β and IL-18 from supernatants of LPS/ATP-stimulated macrophages in the presence of either 3MA (2.5 mM) or BafA1 (75 nM). (**K**) ELISA analysis of IL-1β and IL-18 from supernatants of LPS/ATP-stimulated macrophages ± Rapa (50 nM) in the presence of either 3MA (2.5 mM) or BafA1 (75 nM). Data are representative of more than four independent experiments (Mean + SD). Significance was determined by one-way ANOVA, followed by Tukey's HSD test. ns: not significant, *p < 0.05, **p < 0.01, ***p < 0.001, ****p < 0.0001.

We first examined whether rapamycin inhibits NLRP3 inflammasome activation in macrophages. To address this question, we adopted an established *in vitro* model of NLRP3 inflammasome activation, in which adenosine triphosphate (ATP) drives cleavage of caspase-1 in lipopolysaccharide (LPS)-primed macrophages [9, 19–21]. Macrophages differentiated from THP-1 cells were treated with LPS and then stimulated with ATP in the presence of various concentrations of rapamycin. As expected, the cleaved caspase-1 and secreted IL-1β were markedly increased in macrophages stimulated with LPS/ATP as assayed by Western blot and ELISA, confirming the activation of NLRP3 inflammasome (Figure 1A, 1B). Rapamycin treatment significantly suppressed IL-1β secretion and caspase-1 cleavage in a dose-dependent manner (Figure 1A, 1B). The level of pro-caspase-1 was not reduced by rapamycin (Supplementary Figure 1). To rule out the possibility that the reduction in IL-1β secretion and cleaved caspase-1 resulted indirectly from the cytotoxicity of rapamycin on macrophages, the cell viability was assessed after rapamycin treatment. Rapamycin did not exhibit any cytotoxic effects on the cells as measured by MTT (3-(4,5-dimethylthiazol-2-yl)-2,5-diphenyltetrazolium bromide) assay (Figure 1C). Hence, low but effective concentration (50 nM) of rapamycin was used in subsequent experiments.

Next, we evaluated whether the suppression of NLRP3 inflammasome by rapamycin is mediated through autophagy induction. Western blot analysis revealed that the levels of autophagic proteins (LC3-II, beclin 1, and ATG5) were increased in macrophages after stimulation with LPS/ATP (Figure 1D–1G), consistent with previous reports that upon inflammasome stimulation, autophagy is activated in macrophages as a regulatory mechanism [14, 17]. Rapamycin treatment further increased the levels of LC3-II, beclin 1, and ATG5 (Figure 1D–1G). Additionally, rapamycin caused a decrease in the autophagy adaptor p62/SQSTM1 protein in cultures without bafilomycin A1 (BafA1) and accumulation of p62/SQSTM1 in cultures with BafA1 (Figure 1D, 1H). BafA1 blocks the fusion between autophagosomes and lysosomes, and thereby inhibits degradation of proteins including LC3-II and p62/SQSTM1 [22]. Therefore, these results collectively indicate that rapamycin further enhanced autophagy in LPS/ATP-treated macrophages.

To clarify the role of autophagy in suppression of inflammasome, 3-methyl adenine (3-MA) that blocks autophagosome formation [23] was added to the culture. As expected, 3-MA decreased the levels of autophagic proteins in macrophages (Figure 1I). Inhibition of autophagy by 3-MA increased IL-1β and IL-18 secretion in LPS/ATP-treated macrophages, demonstrating the up-regulation of NLRP3 inflammasome activation (Figure 1J). Importantly, 3-MA reversed the effects of rapamycin in suppressing IL-1β and IL-18 secretion that was significantly increased in LPS/ATP-treated macrophages as a consequence of NLRP3 inflammasome activation (Figure 1K). In addition, when autophagy was suppressed by BafA1 that inhibits the late-phase autophagy [22], similar results were observed (Figure 1J, 1K).

Therefore, the data suggest that autophagy serves as a cell-intrinsic mechanism to limit NLRP3 inflammasome activation, and rapamycin potentiates this regulatory mechanism by inducing autophagy.

### Autophagy induction by rapamycin reduces mitochondrial ROS and pro-IL1β

**Figure 2:**
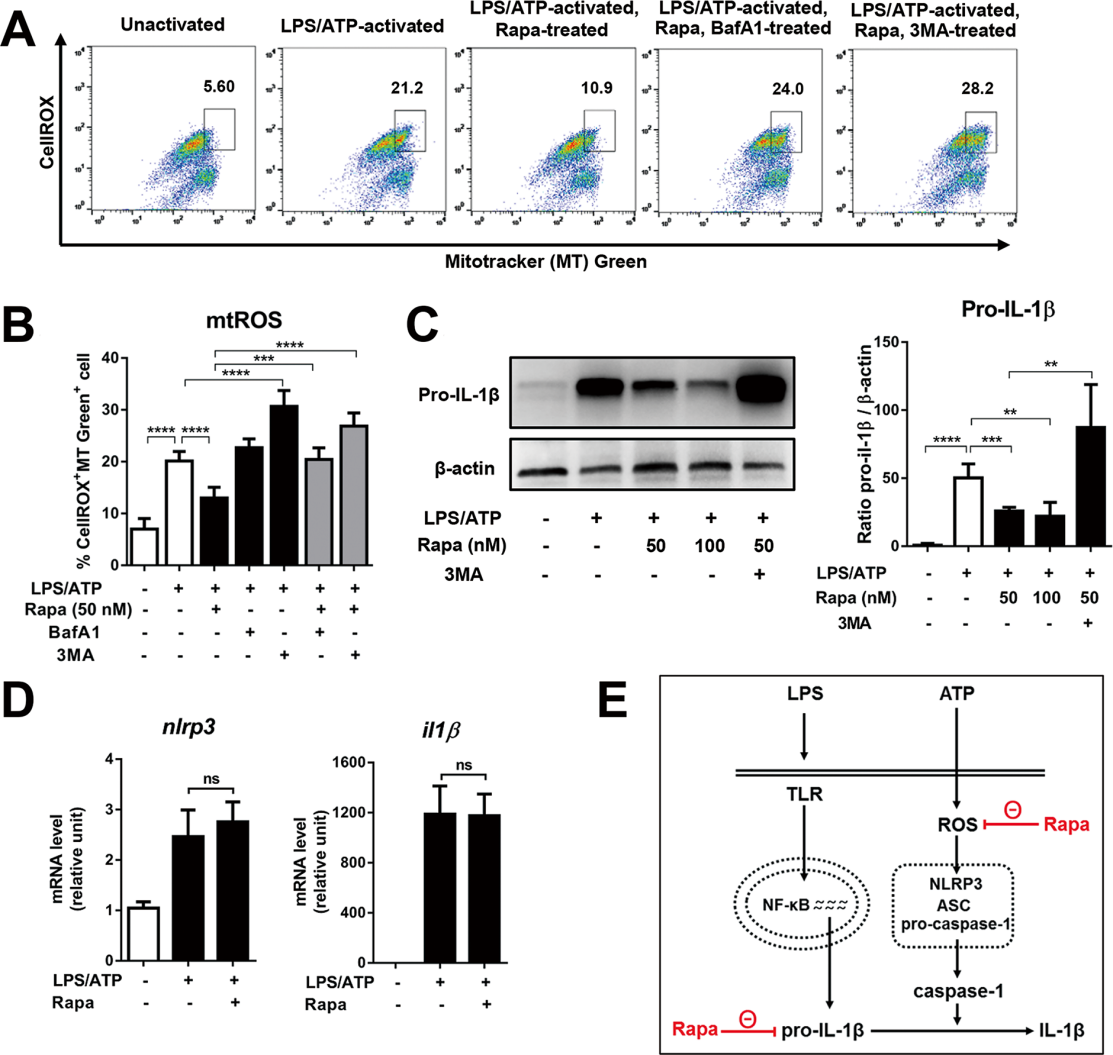
Rapamycin reduces mitochondrial ROS and pro-IL1β, but does not change mRNA levels of NLRP3 and IL-1β Differentiated THP-1 cells were stimulated by LPS (2 μg/mL, 4 h), followed by ATP (5 mM, 45 min) in the presence or absence of rapamycin (Rapa, 50 nM). At 2 h after ATP stimulation, the cells were evaluated for gene transcription or mitochondrial reactive oxygen species (mtROS). 18 h later, the cell lysates were analyzed for pro-IL-1β protein. 3-methyl adenine (3MA, 2.5 mM) or bafilomycin A1 (BafA1, 75 nM) was added to some cultures to block early or late phase autophagy. (**A**, **B**) The level of mtROS was measured by flow cytometry as % of cells that were stained with both CellROX dye and MitoTracker (MT) Green dye and represented as % CellROX^+^MT Green^+^ cells. Shown are representative and quantitative flow cytometry results from three separate experiments. (**C**) Representative images and densitometric analysis of Western blotting for pro-IL-1β in cell lysates. (**D**) The mRNA levels of NLRP3 and IL-1β were analyzed by real-time RT-PCR. The fold changes relative to unstimulated macrophages were calculated by the 2^-ΔΔCT^ method. (**E**) Graphic summary of rapamycin-mediated suppression of NLRP3 inflammasome activation. Data are representative of three independent experiments (Mean + SD). Significance was determined by one-way ANOVA, followed by Tukey's HSD test. ns: not significant, **p < 0.01, ***p < 0.001, ****p < 0.0001.

We next investigated how rapamycin-induced autophagy negatively regulates NLRP3 inflammasome activation in macrophages. A common upstream signal for the activation of NLRP3 inflammasome is oxidative stress, which generates ROS from dysregulated mitochondria [9, 20, 21]. Cells eliminate defective mitochondria using a specialized form of autophagy, called mitophagy [24]. Therefore, we hypothesized that autophagy induction by rapamycin inhibits NLRP3 inflammasome activation through reduction of mtROS. To measure mtROS, the cells were co-stained with CellROX dye that fluoresces upon oxidation by ROS and Mitotracker (MT) Green dye that stains total mitochondria regardless of mitochondrial membrane potential [25]. The level of mtROS, as measured by flow cytometry for CellROX^+^MT Green^+^ cells, was increased in macrophages upon LPS/ATP stimulation (Figure 2A, 2B). Rapamycin significantly reduced the level of mtROS (Figure 2A, 2B). On the contrary, treatment of macrophages with autophagy inhibitor, 3-MA or BafA1, markedly increased the mtROS level (Figure 2A, 2B). Of note, the addition of 3-MA or BafA1 to rapamycin-treated cultures negated the effects of rapamycin in suppressing mtROS in macrophages, indicating that the action of rapamycin was dependent on autophagy (Figure 2A, 2B).

A recent report showed that pro-IL-1β can be targeted for autophagic degradation [15]. To address this possibility, we quantified pro-IL-1β in lysates of macrophages stimulated with LPS/ATP in the presence of rapamycin by Western blot. Correlated with the results obtained for secreted IL-1β (Figure 1A), rapamycin treatment dose-dependently decreased the level of pro-IL-1β in macrophages, and blockade of autophagy with 3-MA abolished the rapamycin effect on the pro-IL-1β level (Figure 2C). These findings indicate that rapamycin treatment results in autophagic degradation of pro-IL-1β, which leads to a reduction in secreted IL-1β.

We further checked whether rapamycin contributes to the decrease in IL-1β at the transcriptional level. Rapamycin treatment did not alter the mRNA levels of either IL-1β or NLRP3 as measured by real-time RT-PCR (Figure 2D), implying that rapamycin suppressed the production of mature IL-1β at the post-transcriptional level.

Collectively, these results suggest that autophagy induction by rapamycin inhibits NLRP3 inflammasome activation in macrophages post-transcriptionally by reducing mtROS and pro-IL1β (Figure 2E).

### p62/SQSTM1 is essential for autophagy induction and NLRP3 inflammasome inhibition by rapamycin

**Figure 3:**
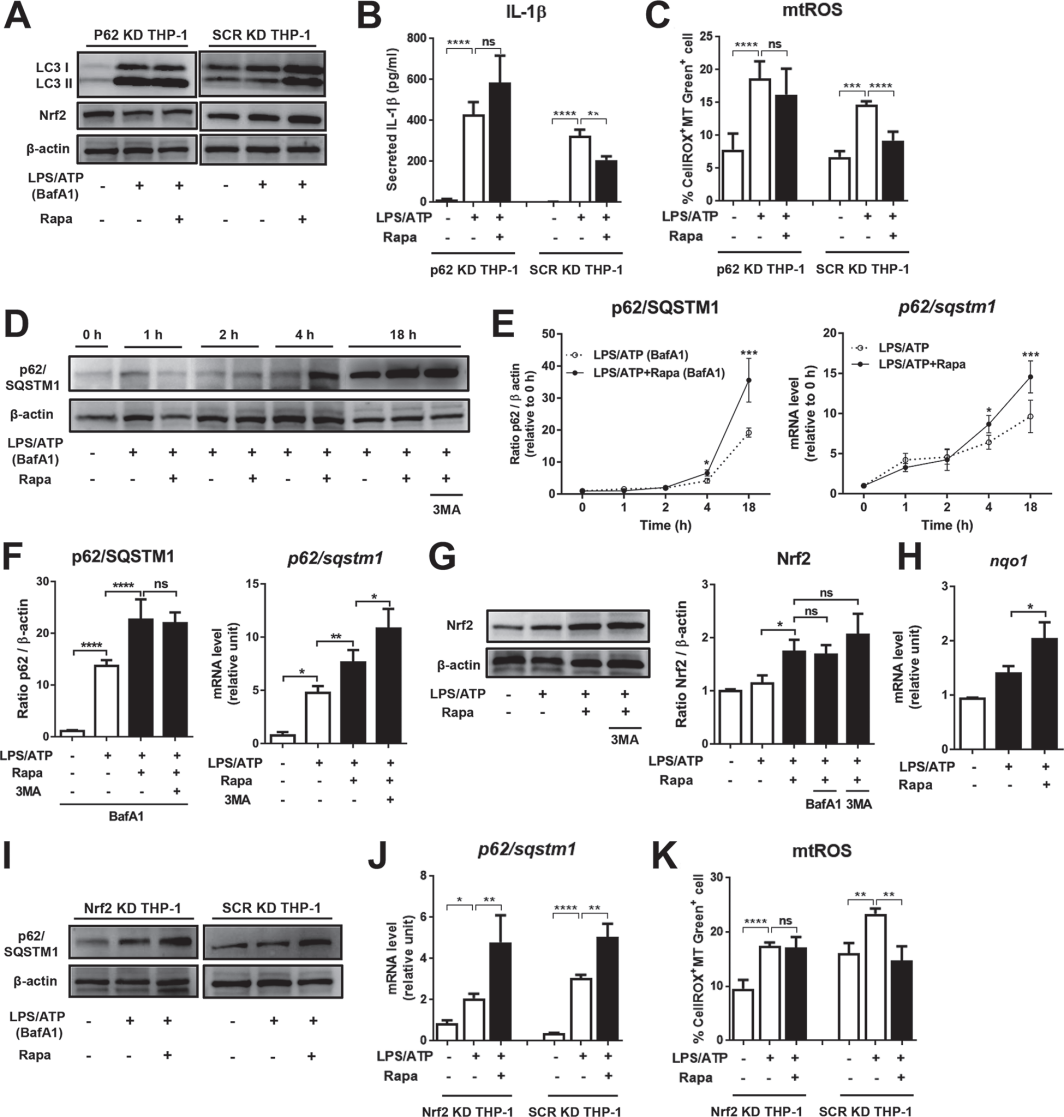
Rapamycin upregulates p62/SQSTM1 and Nrf 2 that are critical for mtROS and NLRP3 inflammasome suppression (**A**–**C**) THP-1-differentiated macrophages were transfected with p62/SQSTM1 (p62 KD THP-1) or control scrambled siRNA (SCR KD THP-1) and stimulated by LPS (2 μg/mL, 4 h) and ATP (5 mM, 45 min) with or without rapamycin (Rapa, 50 nM). The knockdown efficiency of p62/SQSTM1 was confirmed to be 93 ± 0.6% compared to SCR KD THP-1 as assessed by Western blotting and real-time RT-PCR (See Supplementary Figure 2). The protein levels of LC3 and Nrf2 were analyzed by Western blotting in lysates of the cells, and the level of secreted IL-1β was measured by ELISA in the supernatants at 18 h of culture. The mitochondrial reactive oxygen species (mtROS) level was assessed at 2 h by flow cytometry after staining the cells with CellROX and MitoTracker (MT) Green dyes, and presented as % CellROX^+^MT Green^+^ cells. (D-F) Differentiated THP-1 cells were stimulated as in (A) in the presence of bafilomycin A1 (BafA1, 75 nM). 3-methyl adenine (3-MA, 2.5 mM) was added to some cultures to evaluate the autophagy dependency. At indicated time-points, the cells were analyzed for mRNA and protein levels. (**D**) Representative Western blot image of p62/SQSTM1 protein in cell lysates. (**E**) Serial measurements of p62/SQSTM1 protein level by densitometric quantification and of p62/SQSTM1 mRNA level by real-time RT-PCR. (**F**) Quantification of protein and mRNA levels of p62/SQSTM1 in the cells at 18 h. (**G**–**K**) Western blot analysis for Nrf2 and p62/SQSTM1 at 18 h of culture. Real-time RT-PCR assay for Nrf2-target gene NQO1 and p62/SQSTM1 in the cells at 2 h. (I–K) Differentiated THP-1 cells were transfected with either Nrf2 siRNA (Nrf2 KD THP-1) or scrambled siRNA (SCR KD THP-1), and stimulated as in (A). The knockdown efficiency of Nrf2 was 81 ± 0.7% compared to SCR KD THP-1 as assessed by real-time RT-PCR (See Supplementary Figure 2). The mitochondrial reactive oxygen species (mtROS) level as assessed at 2 h by flow cytometry after staining the cells with CellROX and MitoTracker (MT) Green dyes, and presented as % CellROX^+^MT Green^+^ cells. Data are representative of at least three independent experiments and presented as Mean ±/+ SD). Significance was determined by one-way ANOVA, followed by Tukey's HSD test. ns: not significant, *p < 0.05, **p < 0.01, ***p < 0.001, ****p < 0.0001.

Since recent studies showed that ablation of the autophagy adaptor p62/SQSTM1 prevents mitophagy [26] and enhances NLRP3-inflammasome activation [27], we examined whether p62/SQSTM1 is involved in the observed effects of rapamycin. To address this, p62/SQSTM1 was knocked down in macrophages by transfecting siRNA (Supplementary Figure 2), and then stimulated the cells with LPS/ATP in the presence of rapamycin. The knockdown of p62/SQSTM1 completely abrogated the effects of rapamycin on macrophages. Rapamycin did not either enhance autophagy or suppress IL-1β secretion in macrophages with the knockdown of p62/SQSTM1 (Figure 3A, 3B). Similarly, rapamycin was not effective in reducing the mtROS level in macrophages with p62/SQSTM1 knockdown (Figure 3C), supporting the role of p62/SQSTM1 in mitophagy as previously reported [26]. Taken together, data demonstrate that p62/SQSTM1 plays a critical role in mediating the effects of rapamycin on macrophages.

### Rapamycin increases p62/SQSTM1 through transcriptional up-regulation

Previous studies demonstrate that p62/SQSTM1 expression is increased during prolonged starvation in mouse embryonic fibroblasts [28] or by rapamycin treatment in quiescent human fibroblasts [29]. In line with these studies, we observed that the protein level of p62/SQSTM1 was increased by rapamycin in LPS/ATP-stimulated macrophages when assayed in the presence of BafA1 (Figure 1D, 1H). To further investigate the effects of rapamycin on p62/SQSTM1 expression, we performed the time-course study to measure the protein and mRNA levels of p62/SQSTM1 in macrophages following LPS/ATP stimulation with or without rapamycin. To exclude the effect of autophagic degradation of p62/SQSTM1 on the protein measurements, BafA1 was added to the cultures to block p62/SQSTM1 degradation at the lysosome level [22]. Over 18 h following LPS/ATP treatment, both protein and mRNA levels of p62/SQSTM1 gradually increased in macrophages (Figure 3D, 3E). During the first 1 h, the protein levels of p62/SQSTM1 were lower in the rapamycin-treated cells compared to the cells without rapamycin (Figure 3D, 3E). However, from 4 h to 18 h of rapamycin treatment, the levels of p62/SQSTM1 protein were significantly increased by rapamycin treatment (Figure 3D, 3E). The mRNA levels of p62/SQSTM1 were consistently elevated by rapamycin during 18 h of culture (Figure 3E). The increases of p62/SQSTM1 protein and mRNA in rapamycin-treated macrophages were still observed with an addition of 3-MA, showing that the activity of rapamycin in up-regulating p62/SQSTM1 was independent of autophagy (Figure 3D, 3F).

### p62/SQSTM1-dependent Nrf2 activation mediates rapamycin activity in suppressing mtROS

Recent evidence has revealed that p62/SQSTM1 is at the interface linking autophagy and oxidative stress signaling [30, 31]. As an autophagy adaptor, p62/SQSTM1 binds to ubiquitinated protein aggregates and delivers them to the autophagosome, promoting selective autophagy. In addition, p62/SQSTM1 has recently emerged as a regulator of Nrf2-Keap1 (Kelch-like ECH-associated protein 1)-ARE (antioxidant response element) axis by competing with the interaction between Nrf2 and Keap1 and activating the transcription factor Nrf2 whose target genes include antioxidant proteins and detoxification enzymes [32–34]. Since we found that rapamycin increased p62/SQSTM1 (Figure 3D–3F), we postulated that rapamycin might facilitate Nrf2 activation. Indeed, rapamycin increased the level of Nrf2 and Nrf2 target gene NQO1 in macrophages regardless of the addition of 3-MA (Figure 3G, 3H). We next examined whether the increase in Nrf2 might be caused by increased expression of p62/SQSTM1. The level of Nrf2 protein was not elevated by rapamycin in macrophages with p62/SQSTM1 knockdown (Figure 3A). As it was reported that Nrf2 induces p62/SQSTM1 transcription upon oxidative stress [35], we also checked whether rapamycin upregulates p62/SQSTM1 through Nrf2 activation. Rapamycin was still effective in increasing both mRNA and protein levels of p62/SQSTM1 in macrophages with Nrf2 knockdown (Figure 3I, 3J, Supplementary Figure 2). These data indicate that rapamycin activates Nrf2 in a p62/SQSTM1-dependent manner, but Nrf2 is not responsible for p62/SQSTM1 up-regulation by rapamycin.

We further investigated the effect of Nrf2 on oxidative stress induced by LPS/ATP in macrophages. Remarkably, mtROS levels were unchanged by rapamycin in the cells with Nrf2 knockdown, while mtROS was significantly reduced by rapamycin in control cells (Figure 3K). These findings suggest that Nrf2 at least partly mediates the action of rapamycin in suppressing mtROS in macrophages which were activated to trigger NLRP3 inflammasome.

### Autophagy induction by rapamycin inhibits IL1β-p38 MAPK-NFκB pathway

**Figure 4:**
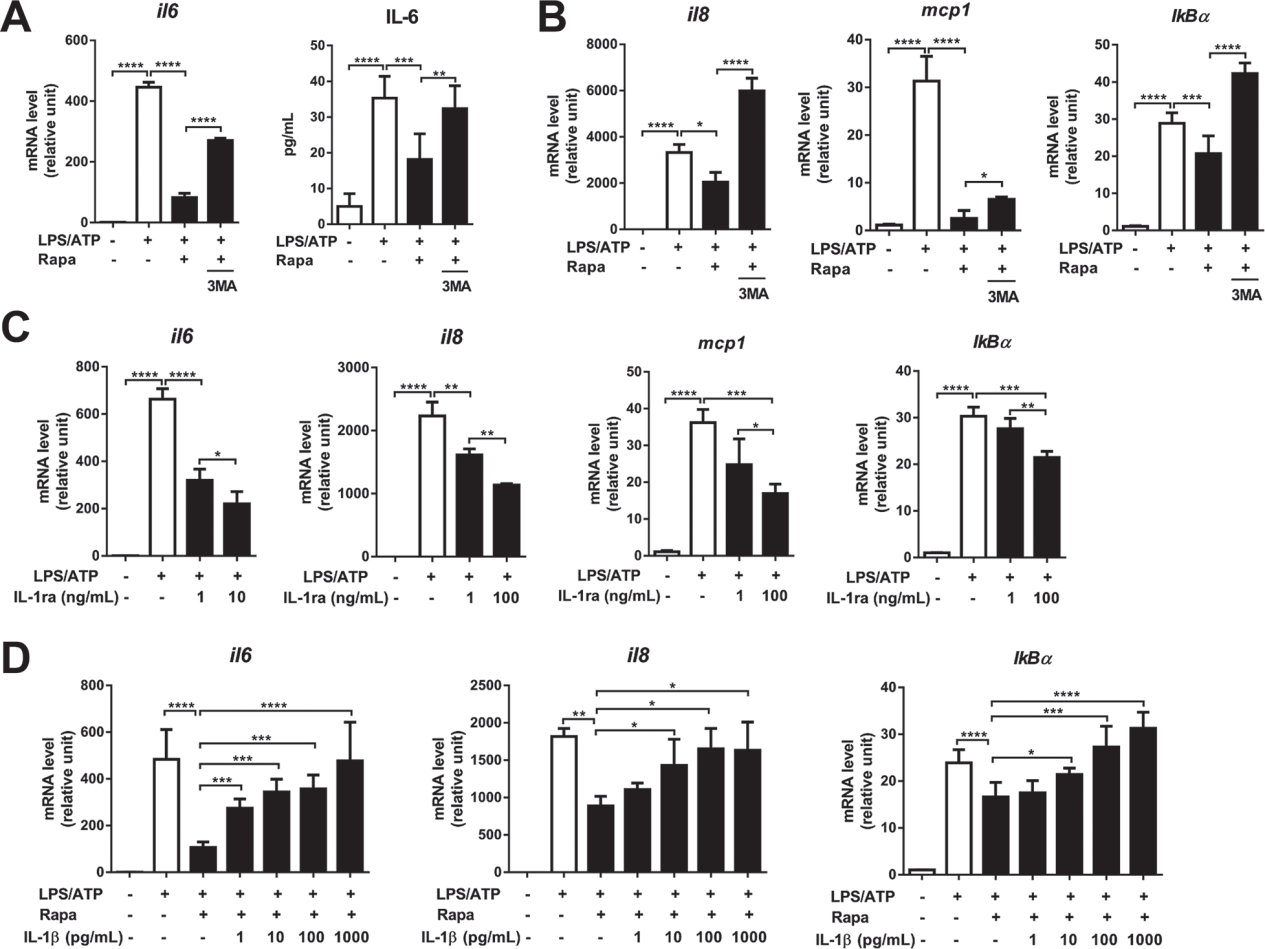
Rapamycin suppresses transcription of IL-6, IL-8, MCP-1, and IκBα Differentiated THP-1 cells were stimulated by LPS (2 μg/mL, 4 h), followed by ATP (5 mM, 45 min) in the presence or absence of rapamycin (Rapa, 50 nM). At 2 h after ATP stimulation, the cells were evaluated for gene transcription, and at 18 h, the supernatants were analyzed for IL-6 protein. (**A**, **B**) The real-time RT-PCR analysis for IL-6, IL-8, MCP-1, and IκBα gene transcription in cell lysates and ELISA analysis for IL-6 protein level in the cell supernatants in the presence or absence of Rapa or 3-methyl adenine (3-MA, 2.5 mM). (**C**) The mRNA levels of IL-6, IL-8, MCP-1, and IκB were analyzed by real-time RT-PCR in LPS/ATP-stimulated cells with addition of IL-1 receptor antagonist (IL-1ra, 0 to 100 ng/mL). (**D**) The mRNA levels of IL-6, IL-8, and IκBα were analyzed by real-time RT-PCR in LPS/ATP-stimulated macrophages ± Rapa with the addition of recombinant human IL-1β at various concentrations (0 to 1000 pg/mL). Data are representative of three independent experiments and presented as the fold changes relative to unstimulated macrophages (Mean + SD). Significance was determined by one-way ANOVA, followed by Tukey's HSD test. ns: not significant, *p < 0.05, **p < 0.01, ***p < 0.001, ****p < 0.0001.

It is well-known that the production of IL-1β by macrophages is essential for initiating and driving inflammatory responses upon tissue injury [36]. Since we found above that rapamycin substantially suppresses IL-1β secretion in macrophages, we further sought to investigate the impact of rapamycin on IL-1β-dependent inflammation. First, we examined how rapamycin affects the transcription of pro-inflammatory genes that are induced in macrophages in response to LPS. As expected, the mRNA levels of IL-1β, TNF-α, IL-6, IL-8, MCP-1 (monocyte chemoattractant protein-1), and IκBα were all elevated in macrophages following LPS/ATP stimulation (Figure 2D, Figure 4A, 4B, Supplementary Figure 3). Rapamycin treatment did not alter the mRNA levels of IL-1β and TNF-α (Figure 2D, Supplementary Figure 3), but markedly reduced the levels of IL-6, IL-8, MCP-1, and IκBα transcripts (Figure 4A, 4B, Supplementary Figure 3). Consistent with the mRNA level, rapamycin significantly decreased the secretion of IL-6 (Figure 4A), and the addition of 3-MA reversed the rapamycin effect on IL-6, IL-8, MCP-1, and IκBα (Figure 4A, 4B). However, rapamycin did not affect the secretion of other inflammation-related cytokines such as TNF-α, IL-2, IL-4, IL-5, IL-10, IL-12, or IL-13 in LPS/ATP-treated macrophages (Supplementary Figure 4).

IL-6, IL-8, MCP-1, and IκBα are canonical IL-1 target genes that are induced in response to binding of IL-1 to IL-1 receptor (IL-1R) [37]. Thus we hypothesized that rapamycin inhibits the levels of IL-6, IL-8, MCP-1, and IκBα in macrophages by reducing IL-1β secretion. In order to check whether extracellular IL-1β is responsible for up-regulation of IL-6 in LPS/ATP-stimulated macrophages, we incubated the cells with IL-1 receptor antagonist (IL-1ra) at various concentrations. The addition of IL-1ra decreased the transcript levels of IL-6, IL-8, MCP-1, and IκBα in a dose-dependent manner (Figure 4C), indicating that secreted IL-1β following NLRP3 inflammasome activation stimulates the gene expression of inflammatory cytokines in LPS/ATP-treated macrophages. Moreover, the addition of recombinant IL-1β dose-dependently increased the transcription of IL-6, IL-8, and IκBα in the rapamycin-treated cells (Figure 4D). Taken together, these data suggest that rapamycin down-regulates the transcription of IL-6, IL-8, MCP-1, and IκBα in LPS/ATP-stimulated macrophages through autophagy-dependent suppression of IL-1β secretion.

**Figure 5:**
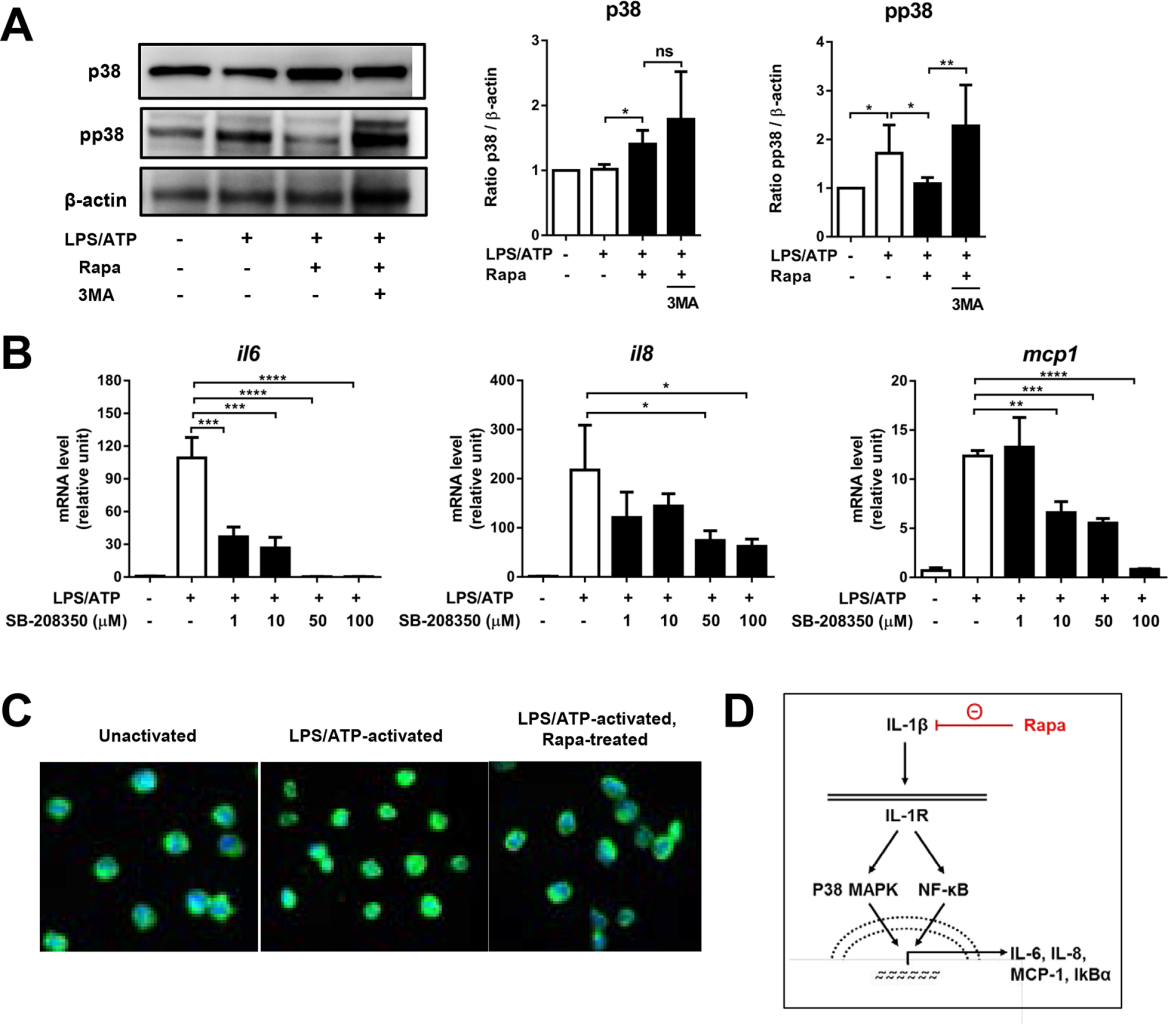
Rapamycin inhibits p38 MAPK-NFκB pathway THP-1-differentiated macrophages were stimulated by LPS (2 μg/mL, 4 h), followed by ATP (5 mM, 45 min) in the presence or absence of rapamycin (Rapa, 50 nM) or 3-methyl adenine (3-MA, 2.5 mM). At 2 h after ATP stimulation, the cells were assayed. (**A**) Representative Western blots of p38 MAP kinase (p38) and phospho-p38 (pp38) in cell lysates. Densitometric analysis presents the ratio of p38 or pp38 relative to β-actin (Mean + SD). (**B**) The real-time RT-PCR analysis for IL-6, IL-8, and MCP-1 transcript levels in LPS/ATP-stimulated cells with addition of p38 inhibitor (SB-208350, 0 to 100 μM). Data are presented as the fold changes relative to unstimulated macrophages (Mean + SD). (**C**) The NF-κB immunofluorescence of the cells. Green (anti-NF-κB) staining indicates NF-κB distribution, and blue DAPI for nuclear staining. (**D**) Graphic summary of rapamycin-mediated suppression of IL1β-p38 MAPK-NFκB pathway. Data are representative of three independent experiments. Significance was determined by one-way ANOVA, followed by Tukey's HSD test. ns: not significant, *p < 0.05, **p < 0.01, ***p < 0.001, ****p < 0.0001.

The NF-κB signaling and p38 MAPK pathways are activated upon IL-1/IL-1R binding and cooperatively induce the expression of IL-6, IL-8, MCP-1, and IκBα [37]. Also, it was previously shown that a positive feedback loop of IL-1 and NF-κB promotes the production of IL-6 in skeletal muscle cells or senescent fibroblasts [38, 39]. Based on this knowledge, we explored the possibility that rapamycin down-regulates the expression of IL-1-inducible genes in macrophages via the p38 MAPK and NFκB signaling. The protein level of phospho-p38 MAPK (pp38) was elevated in macrophages following LPS/ATP stimulation, indicating the activation of the p38 MAPK pathway (Figure 5A). Rapamycin treatment significantly reduced the pp38 level, and the addition of 3-MA abrogated the effect of rapamycin (Figure 5A). Also, inhibition of the p38 MAPK pathway with the specific p38 inhibitor SB-208350 significantly reduced the mRNA levels of IL-6, IL-8, and MCP-1 in LPS/ATP-stimulated macrophages, implying that the IL-1β-induced expression of inflammatory cytokines was dependent on the p38 MAPK signaling pathway (Figure 5B). In addition, rapamycin decreased the translocation of NF-κB from the cytoplasm to the nucleus in LPS/ATP-stimulated macrophages as assayed by immunocytochemistry (Figure 5C). Therefore, the results demonstrate that autophagy induction by rapamycin represses the expression of IL-6, IL-8, MCP-1, and IκBα at a transcriptional level by interfering with the IL1β-p38 MAPK-NFκB feedback loop (Figure 5D).

## DISCUSSION

**Figure 6:**
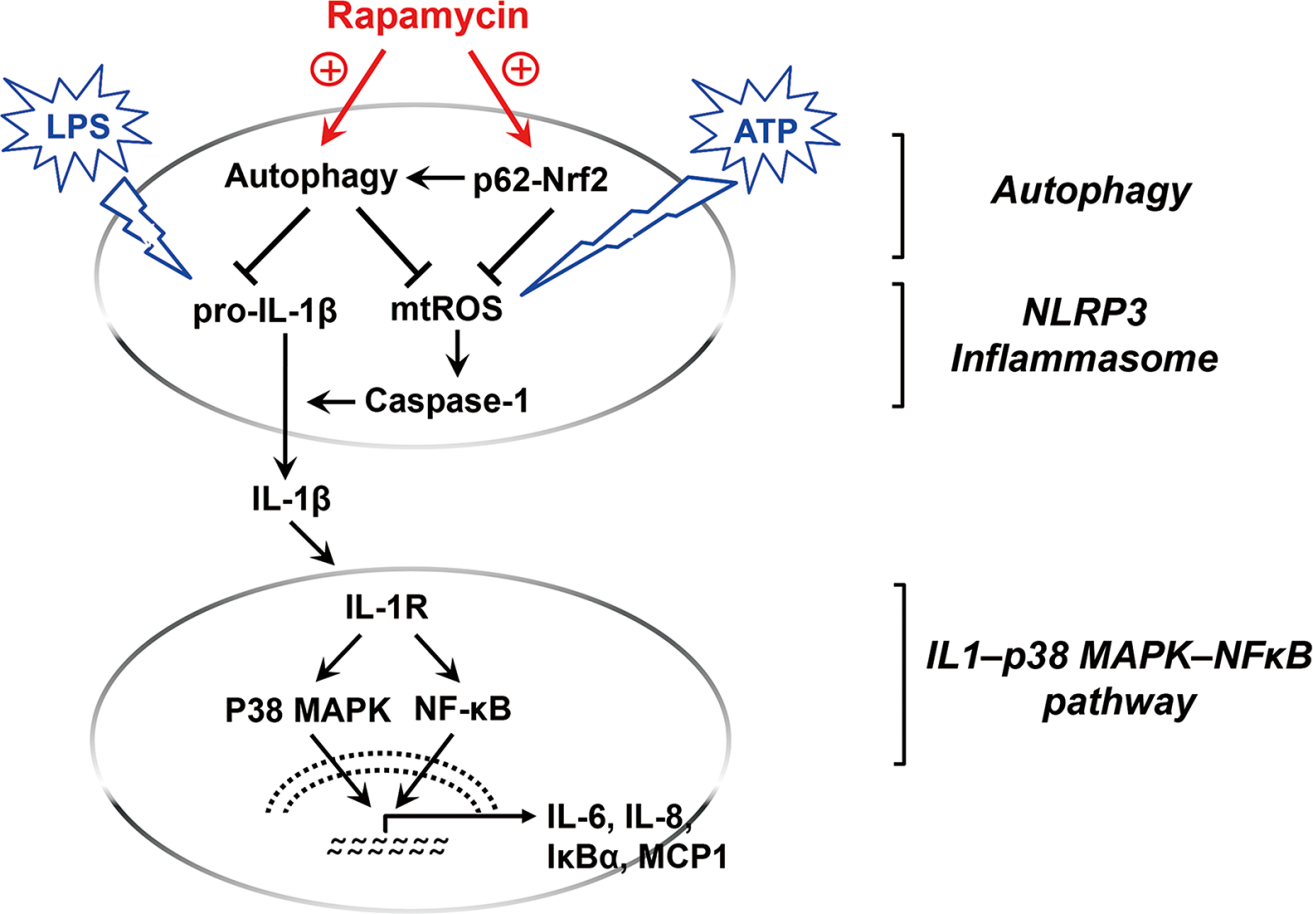
Graphic summary of rapamycin-mediated macrophage modulation Upon activation by LPS and ATP, IL-1β mRNA, pro-IL-1β protein, and mitochondrial reactive oxygen species (mtROS) are increased inside macrophages, which cooperatively elicit the cleavage of caspase-1 into active form and secrete IL-1β outside the cells, i.e. NLRP3 inflammasome activation. Rapamycin induces autophagy, and thus facilitates the autophagic removal of pro-IL-1β and mtROS. Additionally, rapamycin causes transcriptional up-regulation of p62/SQSTM1 which potentiates autophagy and activates Nrf2 pathway to further suppress mtROS. The decreased IL-1β secretion by rapamycin subsequently reduces activation of IL1-p38 MAPK-NFκB pathway, leading to down-regulation of IL-6, IL-8, IκB, and MCP-1.

Our data demonstrate that rapamycin regulates macrophage activation 1) by potentiating a negative regulatory loop between autophagy and NLRP3 inflammasome and facilitating Nrf2-antioxidant pathway in a p62/SQSTM1-dependent manner and 2) by inhibiting a positive feedback loop of IL1-p38 MAPK-NFκB pathways (Figure 6).

Rapamycin, the prototypical inhibitor of mTOR, was originally discovered in soil from Easter Island (locally known as Rapa Nui) as an anti-fungal agent in the 1970s. Since it is increasingly recognized that the mTOR pathway has manifold functions as a central regulator of cell metabolism [6, 40], the mTOR inhibitors including rapamycin are in clinical use as an immunosuppressive treatment to prevent organ transplant rejection [41–43] and stent restenosis [44, 45] or as an anti-cancer therapy [46, 47]. In addition, rapamycin, as a strong autophagy inducer, has been shown to increase lifespan of mice [48] and cultured human fibroblasts [29]. However, it is also reported that inhibition of the mTOR pathway with rapamycin has immunostimulatory effects on blood leukocytes from transplant recipients [49, 50] or on mouse myeloid cells after bacterial infection [51, 52]. Given these multiple and contradictory roles of mTOR pathway, it is important to understand the effects of rapamycin depending on the type of cell and its extracellular and intracellular signals for successful application of rapamycin and its analogs in clinic. In this study, we investigated the effects of rapamycin on human monocyte-derived macrophages activated by NLRP3 inflammasome invoking signals.

Monocytes and macrophages are immune cells that first respond to tissue injury by detecting ‘danger’ signals and initiating the inflammatory process [2, 53]. Prominent among inflammatory pathways in monocytes/macrophages are caspase-1-activating platforms called “inflammasomes” that control maturation and secretion of interleukins such as IL-1β and IL-18, whose potent pro-inflammatory activities direct host response to infection and injury [13]. The NLRP3 inflammasome is the inflammasome best characterized to date, and a tight regulation of NLRP3 inflammasome is essential for preventing long-lasting tissue inflammation and collateral damage as reflected by strong associations between a number of human diseases and dysregulated inflammasome activity [10, 54]. There are cell-autonomous regulatory feedback loops to regulate NLRP3 inflammasome, the most predominant of which is regulation by autophagy. Previous studies have demonstrated that induction of autophagy by inflammatory signals limits NLRP3 inflammasome activation by removing damaged mitochondria and preventing mtROS release [9, 10, 16]. Also, autophagy has been shown to be involved in elimination of ubiquitinated inflammasomes [14] or pro-IL-1β molecules [15]. Consistent with these reports, our results indicate that autophagy induction by rapamycin is effective in suppressing NLRP3 inflammasome activation through the reduction of mtROS and pro-IL-1β.

One interesting finding in our study was that rapamycin upregulated the expression of p62/SQSTM1 in LPS/ATP-treated macrophages, and p62/SQSTM1 played a key role in mediating the rapamycin action in NLRP3 inflammasome suppression. An autophagy adaptor, p62/SQSTM1 has been recently identified as a signaling link that allows the cell to restrain activation of inflammasomes. A recent study by Zhong et al. revealed that NF-κB upregulates p62/SQSTM1 expression in LPS-primed macrophages, which in turn induces mitophagic clearance of damaged mitochondria and thereby attenuates NLRP3 inflammasome-dependent IL-1β production [17]. Another study by Liu et al. recently demonstrated that tripartite motif 11 (TRIM11) interacts with p62/SQSTM1 to degrade AIM2 inflammasome via selective autophagy [55]. Therefore, these findings support the notion that p62/SQSTM1 mediates a negative regulatory mechanism by which macrophages control their own aberrant activation.

Apart from its role in autophagy and NF-κB signaling, p62/SQSTM1 protects the cell against oxidative stress by facilitating the Keap1–NRF2 pathway and activating the protective antioxidant response [56, 57]. In line with this, we found that rapamycin increased Nrf2 activation in a p62/SQSTM1-dependent manner, and Nrf2 mediated the rapamycin activity in reducing mtROS.

The up-regulation of p62/SQSTM1 by rapamycin was previously observed in human fibroblasts [29]. Lerner et al. observed that the mRNA level of p62/SQSTM1 was increased in rapamycin-treated fibroblasts, and the protein level was also elevated by rapamycin starting after 2 h of culture in the presence of lysosomal inhibitors known to block autophagosome. More prominent p62/SQSTM1-positive foci were noted in rapamycin-treated cultures, and p62/SQSTM1 turnover was accelerated by rapamycin. Another study by Sahani et al. showed that prolonged autophagy activation by starvation restored p62/SQSTM1 by transcriptional up-regulation in mouse embryonic fibroblasts and human hepatocellular carcinoma cells, suggesting that the expression level of p62/SQSTM1 does not always inversely correlate with autophagic activity [28]. Further study would be necessary to elucidate the mechanism underlying the elevation in p62/SQSTM1 level we observed in rapamycin-treated macrophages.

Another noteworthy finding from our study was that rapamycin down-regulated the IL1β-p38 MAPK-NFκB pathway in macrophages, leading to reduced levels of inflammatory cytokines including IL-6 and IL-8. Similar to our findings, Laberge et al. reported that rapamycin decreases NF-κB activity and inhibits transcription and secretion of IL-6 and IL-8 in senescent human fibroblasts by reducing IL-1α production [39]. In addition to their roles in inflammation, IL-6 and IL-8 are major components of the so-called senescence-associated secretory phenotype (SASP), a typical feature of senescent cells characterized by the release of various cytokines, growth factors, and proteases [58, 59]. Since the SASP contributes to sterile inflammation which is a hallmark of aging and age-related pathologies, it is possible that the activity of rapamycin to suppress IL-6 and IL-8 might reduce inflammation and “inflammaging” [60] either by preventing the conversion of senescent fibroblasts into pro-inflammatory cells or by repressing excessive activation of immune cells including macrophages.

Macrophages are involved in the pathogenesis of many diseases [4]. Intrinsically, macrophages are well-equipped with multiple mechanisms to control aberrant and prolonged activation. Macrophage modulation by augmenting the cell-intrinsic regulatory mechanisms would represent an attractive strategy to treat or prevent diseases. In that sense, our results might provide a basis for developing potential therapies for diseases that are mediated by NLRP3 inflammasome activation and autophagy impairment in macrophages, such as atherosclerosis [61], diabetes mellitus [62, 63], Crohn's disease [64], Alzheimer's disease [65], uveitis [66] and age-related macular degeneration [67]. The *in vivo* study to explore the clinical relevance of our findings would help substantiate the implication of autophagy-mediated macrophage modulation for the treatment of diseases.

## MATERIALS AND METHODS

### Cells, reagents, and stimulation

THP-1 cells were purchased from American Type Culture Collection (Rockville, MD) and cultured in RPMI (Welgene, Daegu, Korea) supplemented with 10% (vol/vol) heat-inactivated fetal bovine serum (FBS; Gibco, Grand Island, NY) and 1% penicillin-streptomycin (PS; Lonza, Basel, Switzerland) at 37°C in 5% CO_2_. THP-1 cells were differentiated into macrophages by treatment for 3 h with 300 ng/mL phorbol 12-myristate 13-acetate (PMA; Sigma-Aldrich, St. Louis, MO). 24h later, the cells were stimulated to activate NLRP3 inflammasome.

For NLRP3 inflammasome stimulation, THP-1-differentiated macrophages were primed with 2 μg/mL LPS (Ultra-pure LPS, InvivoGen, San Diego, CA) for 4 h, and then treated with 5 mM ATP (InvivoGen) for 45 min. After the cells were washed with phosphate buffered solution (PBS) three times washing, the cells were cultured in high glucose DMEM (Welgene) with 2% (vol/vol) heat-inactivated FBS (Gibco) and 1% PS (Lonza) at 37°C in 5% CO_2_ until further assays.

For treatment, rapamycin (1 to 1000 nM; Sigma-Aldrich), BafA1 (75 nM; Sigma-Aldrich), 3-MA (2.5 mM; Sigma-Aldrich), IL-1ra (1 to 200 ng/mL; Sigma-Aldrich), recombinant human IL-1β (1 to 1000 pg/mL; Sigma-Aldrich), or SB-208350 (1 to 100 μM; Sigma-Aldrich) were added to the cultures simultaneously with LPS priming step and maintained until assays.

### RNA-mediated interference

Differentiated THP-1 cells were cultured in RPMI (Welgene) with 10% (vol/vol) heat-inactivated FBS (Gibco) and 1% PS (Lonza) for 24 h. The cells were then transfected with siRNA for p62/SQSTM1 or Nrf2 (Santa Cruz Biotechnology, CA, USA) or with control siRNA having scrambled sequence (Santa Cruz Biotechnology, CA, USA) using a SG cell line 4D-Nucleofector X kit (Lonza) per the manufacturer's instructions, and incubated for 4 h at 37°C. The degree of gene knockdown was determined by real-time RT-PCR and Western blotting after 24 h of transfection, the same time-point when macrophages were stimulated with LPS/ATP. The knockdown efficiencies of p62/SQSTM1 and Nrf2 were 93 ± 0.6% and 81 ± 0.7%, respectively (Supplementary Figure 1).

### Real-time RT-PCR

For RNA extraction, the cells were lysed in RNA isolation reagent (RNA Bee, Tel-Test Inc., Friendswood, TX) and homogenized with an ultrasound sonicator (Ultrasonic Processor, Cole Parmer Instruments, Vernon Hills, IL). Total RNA was extracted using RNeasy Mini kit (Qiagen, Valencia, CA). After the amount of RNA was measured using Nanodrop 1000 spectrophotometer (Thermo scientific, Waltham, MA), 1 μg RNA was used to generate cDNA by reverse transcription (High Capacity RNA-to-cDNA Kit, Applied Biosystems, Carlsbad, CA). Real-time amplification was performed using TaqMan^®^ Universal PCR Master Mix (Applied Biosystems) in ABI 7500 Real Time PCR System (Applied Biosystems) for the following molecules: NLRP3, IL-1β, IL-6, IL-8, TNF-α, MCP-1, IκBα, p62/SQSTM1, Nrf2 and NQO1 (NAD(P)H dehydrogenase quinone 1). Human PCR probe sets were commercially purchased (TaqMan^®^ Gene Expression Assay Kits, Applied Biosystems). Values were normalized to 18s RNA and expressed as fold changes relative to controls.

### MTT assay

The cell viability was measured using MTT assay (Cell Counting Kit-8, Dojindo Laboratories, Kumamoto, Japan) as per the manufacturer's protocol.

### Western blot analysis

For protein extraction, the cells were sonicated on ice in RIPA Buffer (Biosesang, Seongnam, Korea) containing a protease inhibitor cocktail. After centrifugation at 12,000 rpm at 4°C for 20 min, clear cell lysates were measured for protein concentration by Bradford assay. A total of 20 μg protein was fractionated by SDS-PAGE on 8–16% Tris- glycine gel (Komabiotech, Seoul, Korea), transferred to nitrocellulose membrane (Invitrogen), and then blotted with antibodies against LC3 II (1:500), p62/SQSTM1 (1:4000), beclin 1 (1:10000), ATG5 (1:500), Nrf2 (1:1000) (Novus biological, Littleton, USA), pro-IL-1β (1:1000), p38 (1:1000), phospho-p38 (pp38, 1:1000) (Cell Signaling Technology, Danvers, MA), or β–actin (1:200, Santa Cruz Biotechnology).

### ELISA

The cell-free supernatants were collected from cell cultures after centrifugation at 1500 rpm for 5 min at 20°C, and assayed for concentrations of human IL-1β and IL-6 using ELISA kits (R&D Systems, Minneapolis, MN)

### Multiplex cytokine assay

The cell-free supernatants were assayed by Multiplex Luminex^®^ Assays (Luminex 200 multi-protein analyzer, Luminex, Austin, TX) for the levels of IL-2, IL-4, IL-5, IL-10, IL-12, IL-13, IFN-γ, TNF-α, and GM-CSF.

### Flow cytometry

For cellular and mitochondrial ROS measurements, the cells were stained with both CellROX dye (5 μM; CellROX™ Deep Red Reagent, Invitrogen) and MitoTracker (MT) Green dye (100 nM; MitoTracker Green FM Dye, Invitrogen) at 37°C for 30 min, and analyzed for fluorescence using S1000EXi Flow Cytometer (Stratedigm, San Jose, CA). The data were analyzed using Flowjo software (Tree Star, Ashland, OR).

### Immunofluorescent staining

For NF-κB translocation assay, THP-1-differentiated macrophages were stimulated with LPS/ATP in the presence or absence of rapamycin as mentioned above. After PBS washing twice, the cells were fixed with 100% methanol for 5 min and incubated with 1 μg/mL of anti-NF-κB p65 antibody (ab16502, Abcam, Cambridge, MA) in blocking buffer (5% bovine serum albumin in PBS) overnight at 4°C. The samples were incubated for 1 h with the anti-rabbit IgG (1:2,000) (Alexa Fluor® 488, Invitrogen). The slides were visualized with a fluorescent microscopy (BX-61, Olympus, Tokyo, Japan).

### Statistical analysis

Experiments were independently performed at least three times, each experiment with at least three samples per group. GraphPad Software (GraphPad Prism^®^, Inc., La Jolla, CA) was used for statistical tests. Data were analyzed by one-way ANOVA to compare means of three or more groups. Tuckey's Honestly Significant Difference test was used for a follow-up pairwise comparison. The data are presented as the mean +/± SD. Differences were considered significant at *p* < 0.05.

## SUPPLEMENTARY MATERIALS FIGURES



## Abbreviations

ARE: antioxidant response element
ATP: adenosine triphosphate
BafA1: bafilomycin A1
FBS: fetal bovine serum
IL-1R: IL-1 receptor
IL-1ra: IL-1 receptor antagonist
Keap1: Kelch-like ECH-associated protein 1
LPS: lipopolysaccharide
3-MA: 3-methyl adenine
MAPK: MAP kinase
MCP-1: monocyte chemoattractant protein-1
MT: MitoTracker
mtROS: mitochondrial reactive oxygen species
mTOR: mammalian target of rapamycin
mTORC1: mTOR complex 1
MTT: 3-(4,5-dimethylthiazol-2-yl)-2,5-diphenyltetrazolium bromide
Nrf2: nuclear factor erythroid-derived-2-like 2
PBS: phosphate buffered solution
PMA: phorbol 12-myristate 13-acetate
Pp38: phospho-p38 MAPK
PS: penicillin-streptomycin
SASP: senescence-associated secretory phenotype
TRIM11: tripartite motif 11.
