# Supplementary material for: Rapamycin regulates macrophage activation by inhibiting NLRP3 inflammasome-p38 MAPK-NFκB pathways in autophagy- and p62-dependent manners
Source: Oncotarget. 2017 Apr 19;8(25):40817–31. doi: 10.18632/oncotarget.17256 (PMC5522223; doi:10.18632/oncotarget.17256)
Supplement: Supplementary file 1 [file oncotarget-08-40817-s001.pdf]

# Rapamycin regulates macrophage activation by inhibiting NLRP3 inflammasome-p38 MAPK-NFκB pathways in autophagy- and p62-dependent manners

## Supplementary Materials

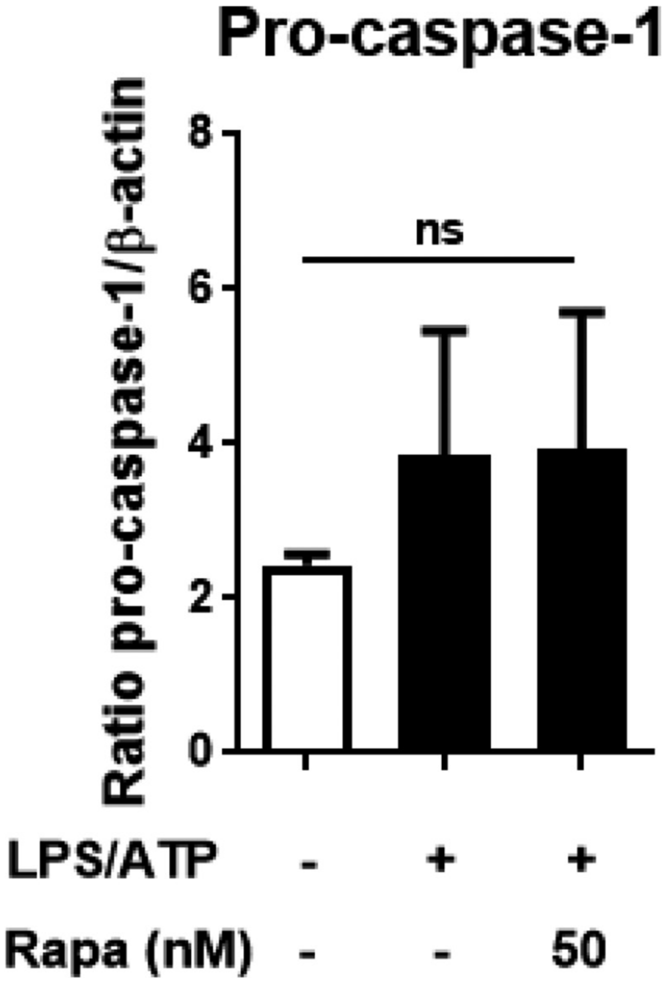


**Supplementary Figure 1: THP-1-differentiated macrophages were stimulated by LPS (2 µg/mL, 4 h), followed by ATP (5 mM, 45 min) in the presence of rapamycin (Rapa, 50 nM).** After 18 h of culture, the cells were analyzed for the pro-casepase-1 level by Western blotting.

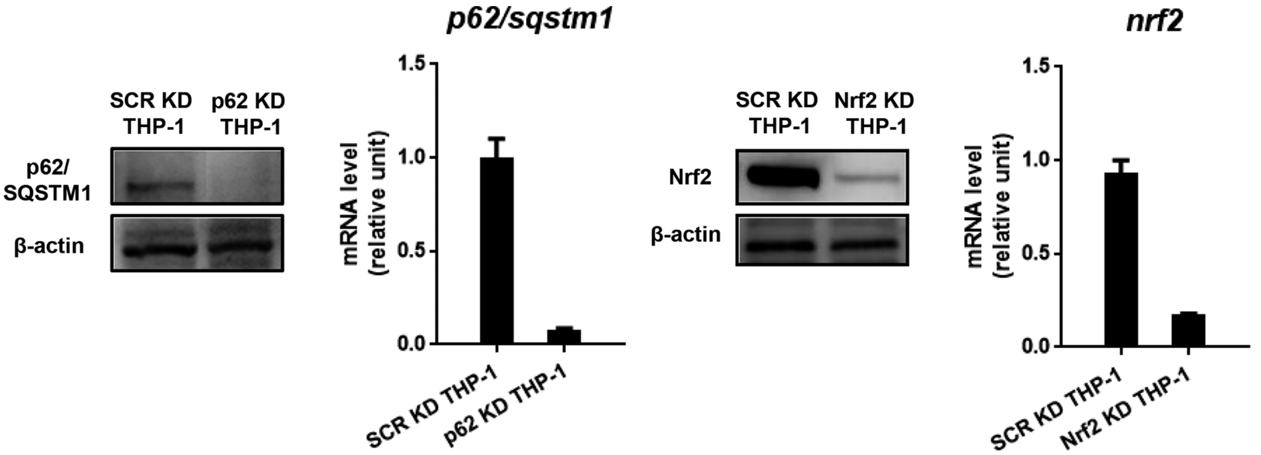


**Supplementary Figure 2:** The knockdown efficiency of p62/SQSTM1 siRNA (p62 KD THP-1) and Nrf2 siRNA transfection (Nrf2 KD THP-1) in macrophages was evaluated at 18 h after the start of transfection by Western blotting and real-time RT-PCR, and found to be 93 ± 0.6% and 81 ± 0.7%, respectively, compared to the cells transfected with control scrambled siRNA (SCR KD THP-1).

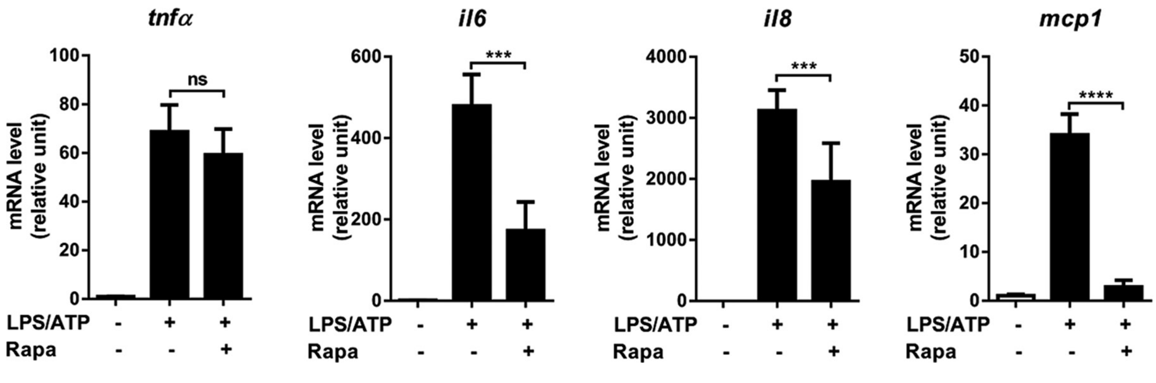


**Supplementary Figure 3: The mRNA levels of TNF-α, IL-6, IL-8, and MCP-1 (monocyte chemoattractant protein-1) in LPS/ATP-stimulated macrophages in the presence or absence of rapamycin (Rapa, 50 nM) as analyzed by real-time RT-PCR.** The fold changes relative to unstimulated macrophages were calculated by the $2^{-\Delta\Delta CT}$ method.

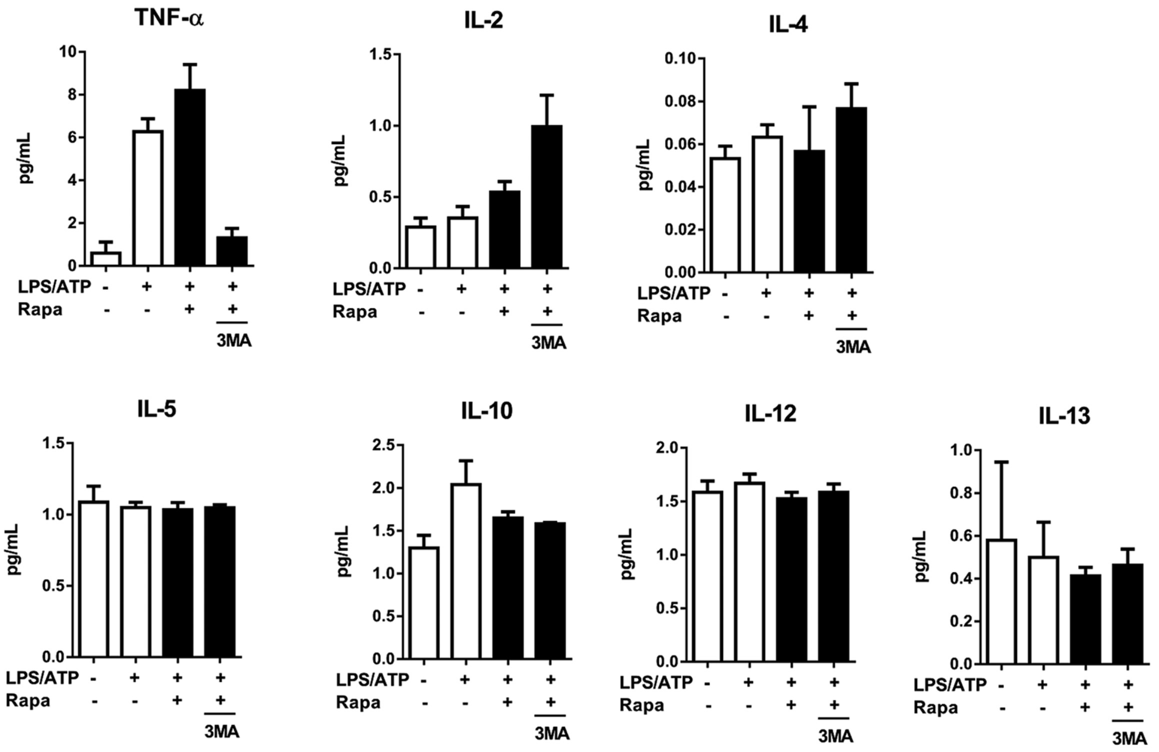


**Supplementary Figure 4: The multiplex cytokine assays in the supernatants from LPS/ATP-stimulated macrophages in the presence or absence of rapamycin (Rapa, 50 nM).** 3-methyl adenine (3-MA, 2.5 mM) was added to evaluate the autophagy dependency of cytokine release. No significant changes in cytokine levels were observed between groups.
